# A Multiaxial Fatigue Damage Model Based on Constant Life Diagrams for Polymer Fiber-Reinforced Laminates

**DOI:** 10.3390/polym14224985

**Published:** 2022-11-17

**Authors:** Aleksandr Elkin, Viktor Gaibel, Dmitry Dzhurinskiy, Ivan Sergeichev

**Affiliations:** Center for Materials Technologies, Skolkovo Institute of Science and Technology, Bolshoy Boulevard 30, Bld. 1, Moscow 121205, Russia

**Keywords:** fiber-reinforced polymer composites, fatigue, damage initiation, residual strength, finite element analysis

## Abstract

In the last decade, fatigue damage models for fiber-reinforced polymer composites have been developed assuming the fracture energy equivalence hypothesis. These models are able to predict a fatigue life of composite laminates, but their identification requires a significant number of off-axial tests for various stress ratios. The present study proposes the stress ratio dependent model, which phenomenologically adopts a decrease in stiffness and residual strength of a unique ply according to experimental constant life diagrams. Hashin, Tsai–Hill, and the maximum stress failure criteria are utilized for damage initiation considering the residual strength of the ply. The obtained results indicate a sufficiency of using S-N curves for UD 0°, UD 45°, and UD 90° for identification of the model. The model was verified by S-N curves for UD 10°, UD 15°, and UD 30° and its applicability was demonstrated for prediction of a fatigue life of composite laminates with an arbitrary lay-up. The model is implemented into ABAQUS finite element software as a user subroutine.

## 1. Introduction

In recent decades, metallic components have been replaced by fiber-reinforced plastics (FRPs) in industry and infrastructure, e.g., glass FRPs are widely used in wind turbine blades [1] and pressurized vessels [2]. FRPs demonstrate better fatigue performance relative to metals, and, in earlier engineering analysis, composite structures were treated even as fatigue free [3]. However, recent studies show that cyclic loads significantly reduce stiffness and strength of FRPs, and these effects cannot be neglected.

Nowadays, a variety of approaches were proposed for prediction of fatigue life of materials and structures under multiaxial stress state. For example, the critical plane-based criteria have been developed to assess fatigue performance of notched and unnotched metal components [4,5,6,7]. Zhu et al. [7] concluded that the modified Smith–Watson–Topper criterion [8] is more suitable for brittle materials, and the modified generalized strain energy criterion [7] provides better results for ductile materials. Nassiraei et al. [9,10] improved fatigue life of composite structures by lay-up optimization for various FRPs to reduce stress concentration factors under bending and axial loads in tubular X-connections utilizing finite element analysis (FEA).

**Figure 1:**
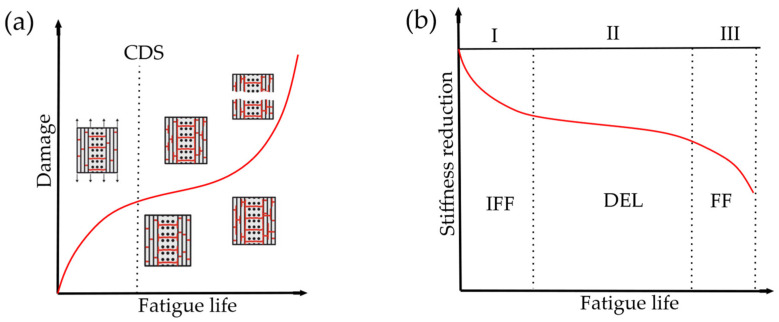
Fatigue failure in multilayered FRPs: (**a**) Formation of cracks, i.e., damage accumulation; (**b**) Stiffness reduction for the described failure stages [14,15].

The fatigue phenomenon in FRPs is divided into three main stages [3,11,12,13], as presented in Figure 1. The first stage characterizes the initiation of small unconnected matrix cracks, causing inter-fiber failure (IFF). The second stage begins when the accumulated cracks form macro damage, so-called characteristic damage state (CDS), such as delamination (DEL) or large inter-layer cracks. The final stage occurs when fibers begin to fail (FF) that causes the failure of the multilayered composite.

**Figure 2:**
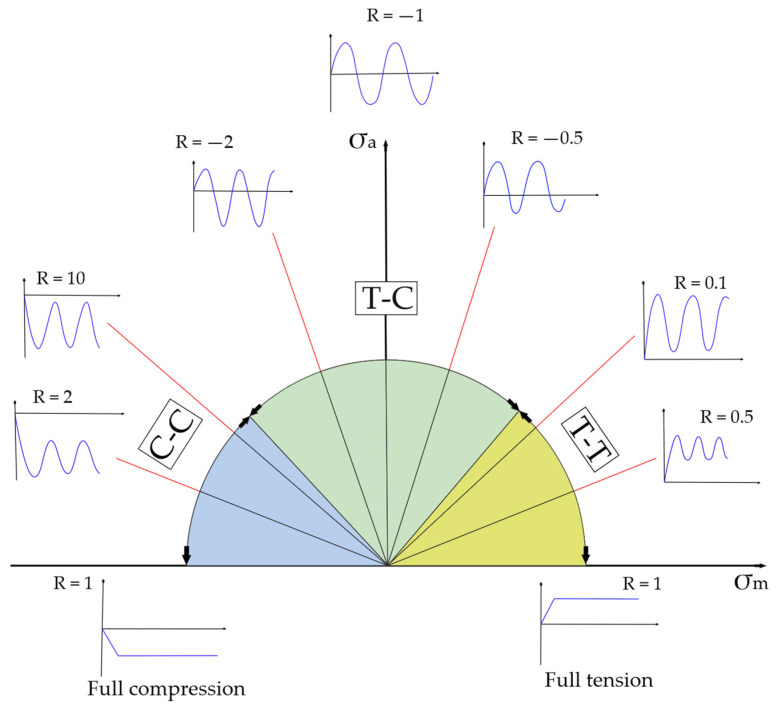
The CLD diagram illustration: A vertical axis—the alternate stress; A horizontal axis—the mean stress [21].

For estimating fatigue life of FRPs, studies perform tests, applying a cyclic load of various magnitudes with a constant stress ratio (R-ratio) to define S-N curves. To understand fatigue behavior under various R-ratios, it was proposed to use constant life diagrams (CLD) (or constant fatigue life diagram) [16,17]. The CLD is the expansion of the Goodman diagram [1,18,19,20], a representation of S-N curves in which the horizontal axis presents a mean stress, and the vertical axis describes the difference between maximum and minimum stress during the loading cycle, so-called alternate stress. Designations of a CLD are given in Figure 2.

Fatigue damage models (FDMs) are widely adopted for analysis due to their capability to predict fatigue life. Most FDMs are phenomenological, and they are identified by fatigue data (S-N curves, residual strength, or stiffness degradation rules). Verified FDMs are able to predict fatigue failure and reveal the failure phenomenon [22]. The FDMs are divided into direct and stepwise models (cumulative damage models). In the direct FDMs, the degradation and fatigue cycles are calculated instantly based on a formulated relationship between material properties and load applied. The authors of [23,24] showed that direct FDMs are able to predict laminate failure based on fatigue properties of unidirectional (UD) laminates. In the stepwise FDMs, degradation accumulates cycle-by-cycle. As usual, such FDMs are utilized in conjunction with FEA.

The degradation of material properties depends on stress state obtained by FEA and input fatigue data. The stepwise approach with FEA allows to build a generalized FDM in which properties degrade in certain elements under various loading conditions. Such FDMs are based on various hypotheses, e.g., the Pfanner’s fracture energy equality hypothesis [25]. Kruger et al. [26] proposed the layer-based FDM in which strength and stiffness decrease according to the Pfanner’s hypothesis. They demonstrated simulation results, but not verified their model. Brod et al. [14] developed and verified their own FDM, predicting fatigue life under an inhomogeneous stress state for cross-ply carbon FRP with the similar hypothesis (static fracture energy equals fatigue fracture energy). They used fatigue properties of UD samples as a lamina property to predict laminate failure that potentially may reduce the total amount of tests required for composites. Most similar FDMs use CLDs to define fatigue life and compute fracture energies for failure predictions. However, it seems that it is possible to analyze fatigue damage without appealing to fracture energies as presented in works [27,28,29].

The main goal of the current study is to present a stress ratio dependent stepwise layer-based fatigue damage model, discussing its capabilities and drawbacks. The objectives of the paper are the following:

- Describe the theory of the proposed FDM, main hypothesis and assumptions;
- Detail FEA implementation features;
- Present the comparison between the FDM predictions and the experimental data, discuss the prediction flaws.

The purpose of the proposed FDM is to minimize static and fatigue tests needed for prediction of the laminate fatigue behavior.

The article is organized as follows: Section 1 introduces the fatigue problem of FRPs, explaining concepts and approaches. Section 2 is divided into two subsections. In the first one, we described the FDMs background, assumptions, hypothesis, and its implementation; the second subsection includes static and fatigue material properties used for the FDM identification and verification. In Section 3, we present the results of the FDM calculations and compared them with experimental fatigue data. Section 4 contains a critical evaluation of the results; factors influencing the outcomes are discussed. In the final section, we conclude the work and provide an outlook on further development of the proposed FDM.

## 2. Materials and Methods

### 2.1. Theoretical Background of the FDM

The proposed FDM was developed based on the hypothesis that stiffness and residual strength reduce according to experimentally determined constant life diagrams. Such an approach allows to predict fatigue life of FRPs by CLDs for UD 0° 45° and 90° (longitudinal, shear and transverse fatigue properties, respectively). It is assumed that residual strength decreases cycle-by-cycle according to input or interpolated S-N curves. The model does not utilize the energy equivalence hypothesis (Pfanner’s hypothesis) that dissipated energies under static and cyclic loading are the same, as presented in works [25,26]. In the proposed FDM, different loading conditions can be applied, e.g., a “load-1” to a structure “number-1” cycles with “stress ratio-1” then a “load-2” to the structure “number-2” cycles with “stress ratio-2” in the same analysis. It is considered that the stiffness and residual strength of the material degrade differently under tension and compression. Every residual strength component has its own degradation variable. Therefore, the complex stress state is taken into account.

The FDM was implemented as a user material subroutine (UMAT) in the ABAQUS finite element software. The FDM is layer-based, and lamina material properties are considered elastic and orthotropic. It was assumed that laminates have no initial defects, such as delamination; thus, the laminate layers are bonded perfectly. The subroutine decreases stiffness and residual strength cycle-by-cycle in every integration point. The implementation of the FDM is demonstrated for 3D hexagonal finite elements.

**Figure 3:**
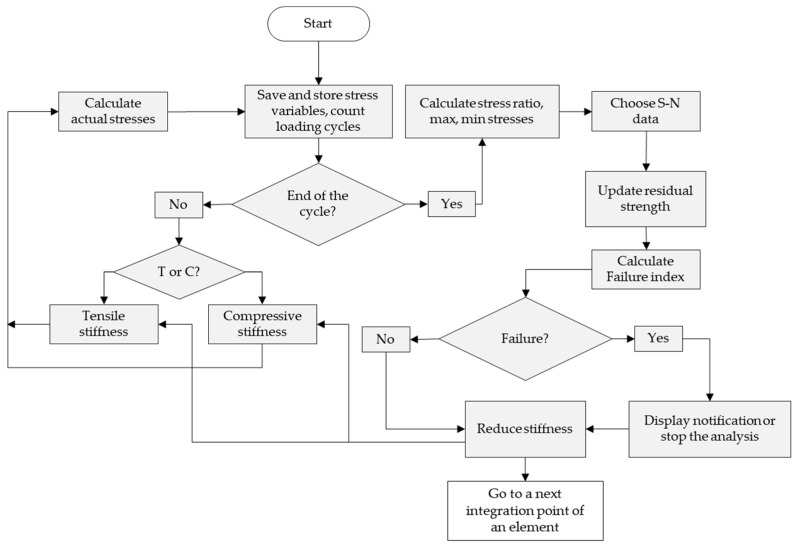
The FDM workflow.

The workflow of the proposed FDM is shown in Figure 3 and is composed of two parts. The first one consists of blocks that determine the load mode (tension or compression) and calculate a stress vector until a fatigue cycle is not completed. The second part includes blocks that determine the residual strength and stiffness degradation. At the end of the loading cycle, the stored variables are used to calculate the maximum and minimum actual stresses to define a stress ratio (at least two-time increments are required for analysis).

**Figure 4:**
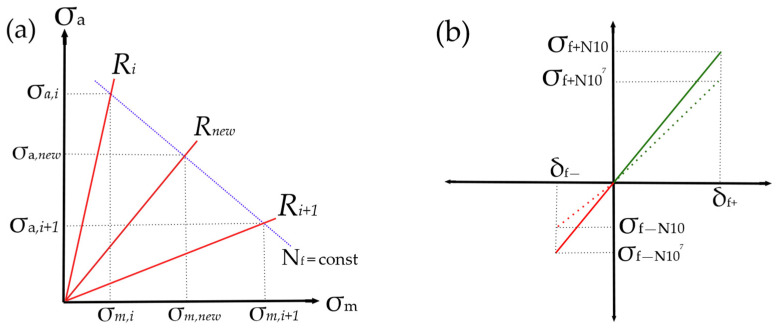
Assumptions accepted: (**a**) Linear interpolation between S-N curves for a predefined R-ratios; (**b**) Proportional to stress stiffness reduction.

Then, according to the input CLD, the subroutine uses the calculated stress ratio to choose the S-N data. As previously proposed [14], unknown S-N curves are linearly interpolated, see Figure 4a. Next, the residual strengths for the current and next loading cycle are calculated, and the degradation increment is determined as a difference between these residual strengths. After that, the residual strength is updated by subtracting of the degradation increment.

The updated residual strength is compared with the actual stress. If the residual strength is less than the actual one, then the stiffness of an element becomes negligibly small (it means element failure). In the next step, a user-defined failure criterion of the element is calculated, in which a multiaxial stress state is considered. If the element is not failed, the material stiffness is decreased by a small fraction which proportional to the strength reduction, as shown in Figure 4b. However, earlier studies demonstrated that material stiffness does not always decrease as strength does [30,31], depending on fiber orientation, fiber and resin types, and stress ratio. Thus, the proposed FDM allows to set the relationship between the stiffness and residual strength according to experimental results.

To avoid issues during numerical computation, the following solutions were employed. A reverse stress ratio (*RR* = σ_max_/σ_min_) was declared as an independent variable in addition to a conventional stress ratio for the ‘left part’ of the CLD when σ_m_ < 0 (see Figure 2) to exclude infinite numbers (*R* = σ_min_/σ_max_ = −∞, if σ_max_ = 0). Since the input S-N curves define fatigue behavior only for certain stress ratios, the linear interpolation was used to calculate S-N curve for intermediate stress ratios. For that, the linear interpolation coefficient k was introduced into the model. To connect k and R for the interpolated S-N curves, the second order polynomial was used, Equation (1). The k values were calculated for various stress ratios to fit the coefficients $a_{1},a_{2},a_{3}$.

(1) $$k=a_{1}R^{2}+a_{2}R+a_{3}.$$

The input S-N curves were approximated using the second order power law, as presented in Equation (3). A similar approach was successfully applied by Palmgren and Miner [32,33].

In addition, the problem of unknown S-N curves for regions of low alternative stress is solved. In these regions, the CLD is defined for *R* = 0.5 or 2, but it is undefined for values close to *R* = 1 (the full tension or compression). Therefore, we assumed that the degradation strength rate for *R* = 1 is ‘large’ and the fitting coefficient B = −2 to drop the residual strength to almost zero after one loading cycle.

### 2.2. Initial Data

The FDM relies on the experimentally determined CLDs, and quantity of required test data is minimized, limiting only three CLDs of UD samples under uniaxial loading with the following fiber orientations:

- Zero-degree-oriented CLD to determine a strength degradation along fibers;
- Forty-five-degree-oriented CLD to determine a degradation of shear strength;
- Ninety-degree-oriented CLD to determine a strength degradation across fibers.

It is believed that chosen CLDs describe fiber and matrix failure modes in FRPs.

**Figure 5:**
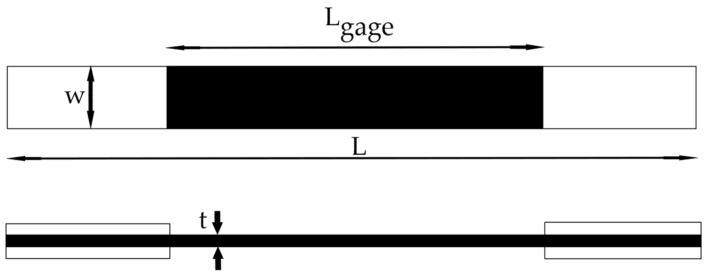
The samples geometry.

Kawai et al. [34] performed comprehensive experimental studies on FRPs UD laminates fabricated from TORAY T700S carbon fibers and 2592 epoxy resin prepreg tapes (T700S/2592) and cured in autoclave. The sample dimensions are presented in Table 1. The geometry is shown in Figure 5. The long samples were used for tension-tension (T-T) fatigue tests; the short ones for tension-compression (T-C) and compression-compression (C-C) fatigue tests.

The material properties are given in Table 2 and Table 3. The fatigue tests were performed at a temperature of ~23 °C with a frequency of 5 Hz. The S-N curves and CLDs were obtained for the following fiber orientations: 0, 10, 15, 30, 45, 90°. The test data were used for identification (UD 0°, 45° and 90°) and verification (UD 10°, 15° and 30°) of the proposed FDM. 

**Figure 6:**
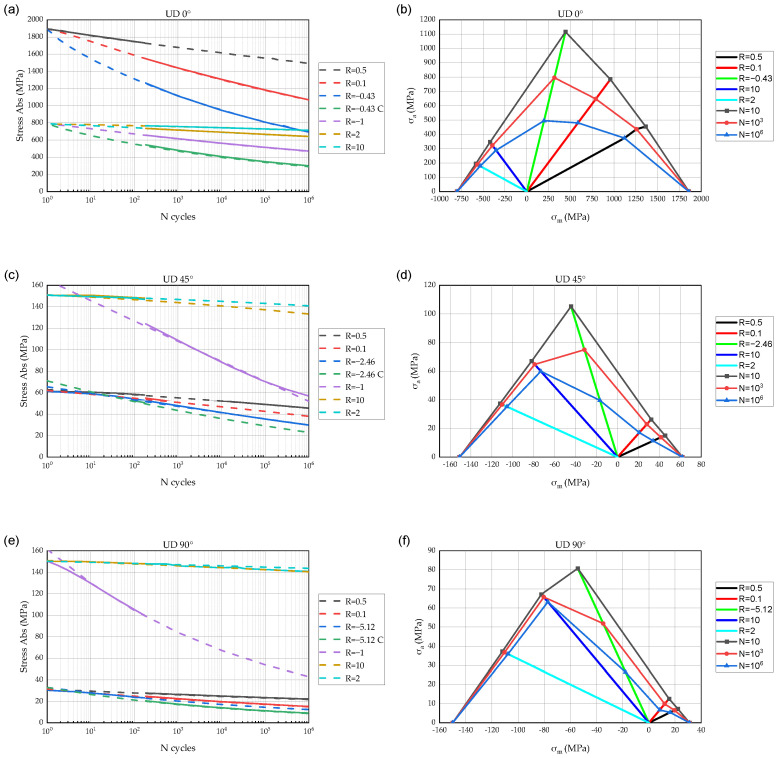
Approximated CLDs and S-N curves based on data [34] used in the FDM as lamina fatigue properties. S-N curves: (**a**) UD 0°, (**c**) UD 45°, (**e**) UD 90°. The CLDs: (**b**) UD 0°, (**d**) UD 45°, (**f**) UD 90°.

The experimental CLDs included at least five points, representing the S-N curves for five different stress ratios which cover the T-T, T-C, and C-C load conditions. The CLDs and S-N curves used in the FDM are presented in Figure 6. On the left side S-N curves are presented. The initial experimental S-N curves have solid lines, and the approximated curves by Equation (3) marked with dash lines. The obtained CLDs for the FDM are presented on the right side of Figure 6.

Kim et al. [37] approximate the fatigue data [34] using Equation (2), but that approach is not applicable to the proposed FDM, because fitting parameters turn out significantly small (~10^−300^) which makes the finite element implementation difficult.

(2) $$\sigma >0:\;\;\;\;N_{f}=\frac{1{\left(R^{t}\right)}^{-\beta }}{\alpha \left(\beta -1\right)}\;\left[\;{\left(\frac{\sigma_{max}}{R^{t}}\right)}^{1-\beta }-1\right]+N_{0},\;\;\sigma <0:\;\;\;\;N_{f}=\frac{1{\left(R^{c}\right)}^{-\beta }}{\alpha \left(\beta -1\right)}\;\left[\;{\left(\frac{\sigma_{min}}{R^{c}}\right)}^{1-\beta }-1\right]+N_{0}.$$

Instead, we propose to use the second order power law to fit the S-N curves and calculate the input CLDs by Equation (3), where $\sigma_{res}$—the absolute residual strength; *A, B*, and *C*—fitting parameters. The sum of *A* and *C* represents initial residual strength (ultimate strength), and B defines the degradation strength rate (or the slope of a S-N curve).

(3) $$\left|\sigma_{res}\right|=AN_{i}^{B}+C.$$

The equation provides a suitable accuracy, and the fitting parameters do not induce computing issues, unlike the S-N curve approximation given by Equation (2). The fitting parameters of the proposed Equation (3) are presented in Table 4. In the table, the R_crit_ equals to the ratio of compressive to tensile strength, providing the fastest strength degradation rate. Due to approximation errors, $\sigma_{res}$ values are larger than initial fatigue stresses for the low cycle fatigue region (see Figure 6).

**Figure 7:**
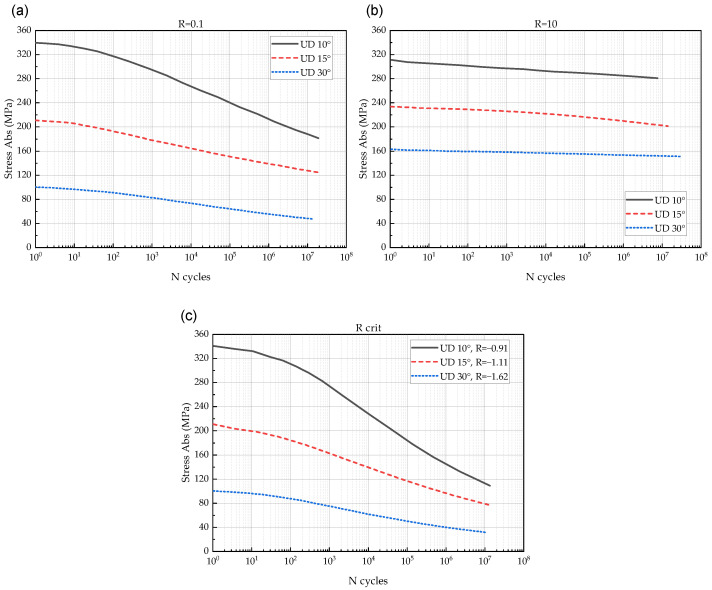
S-N curves used for verification of the FDM [29]: (**a**) UD 10°, (**b**) UD 15°, (**c**) UD 30°.

To verify the proposed FDM, we compared experimental S-N curves for UD 10°, 15°, and 30° as shown in Figure 7 with the curves predicted by the model.

**Figure 8:**
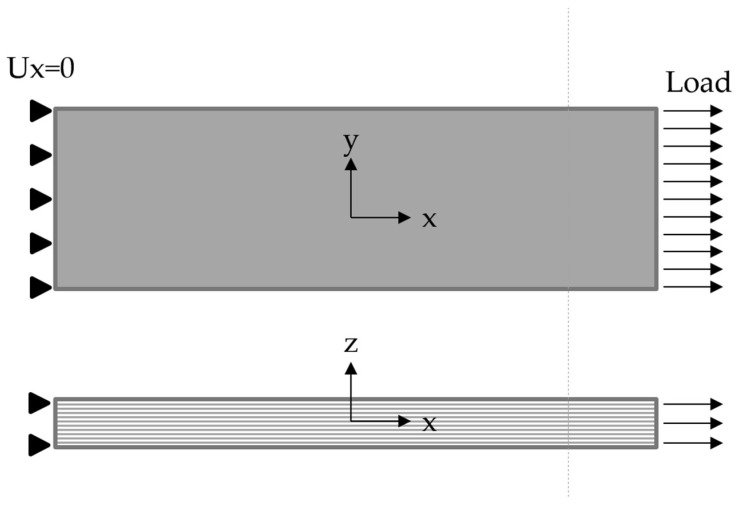
The finite element model for the FDM verification.

The simple ABAQUS FE model was built to estimate the fatigue life of FRP samples with various fiber orientations (see Figure 8). X-axis is a reference axis for composite fiber orientation. Dimensions of the FE model were the same as the dimensions of UD samples shown in Table 1. The C3D8 linear brick elements with a characteristic size of 1 mm were used.

The three conventional failure theories for FRPs, Hashin, Tsai–Hill, and the maximum stress, were adopted (see Table 5). We assumed that failure stress in the criteria is the residual strength that decreases according to the considered degradation law. The criteria were implemented in the FDM.

In Table 5, X, Y—the normal tensile residual strength in longitudinal and transverse directions, respectively; $Y_{C}$—the normal compressive residual strength; $S_{L},\;S_{T}$—the longitudinal and transverse residual shear strength, respectively.

## 3. Results

**Figure 9:**
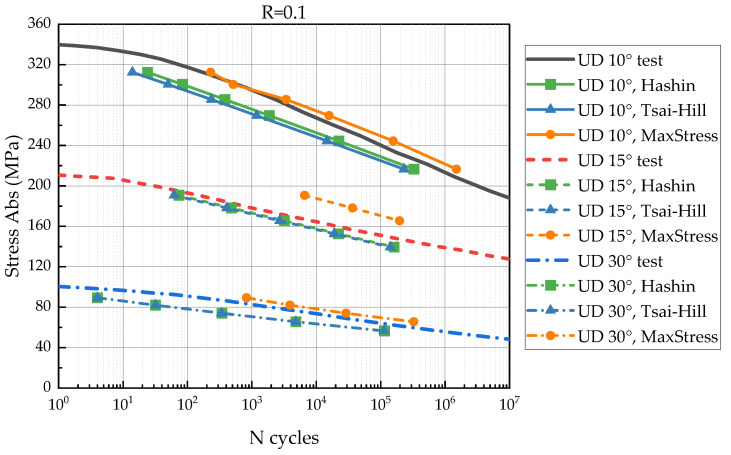
Comparison between experimental and predicted S-N curves of UD 10, 15, 30° for *R* = 0.1.

**Figure 10:**
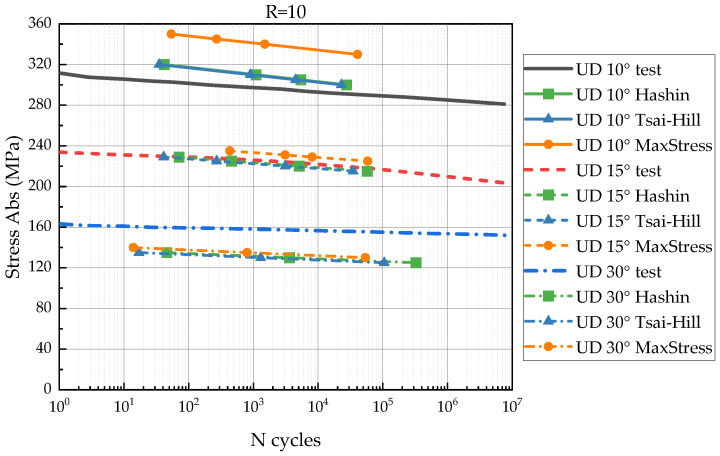
Comparison between experimental and predicted S-N curves of UD 10, 15, 30° for *R* = 10.

**Figure 11:**
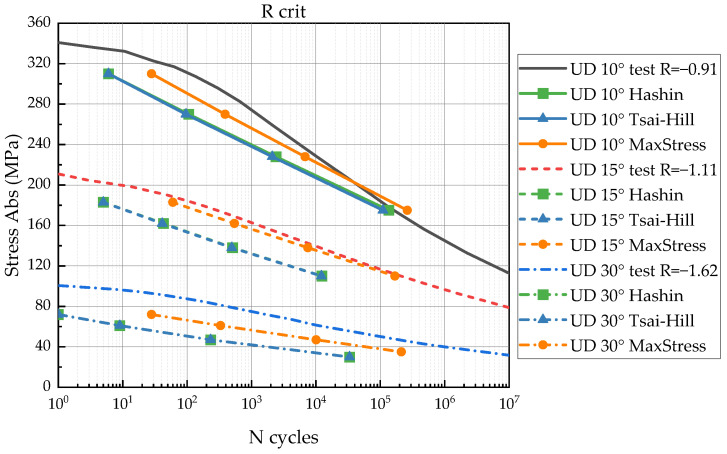
Comparison between experimental and predicted S-N curves of UD 10, 15, 30° for R_crit_ (combined tension-compression mode).

The comparison between experimental S-N curves and predictions is presented in Figure 9, Figure 10 and Figure 11. In the figures, the predicted S-N curves have lines with markers; the experimental S-N curves are shown with solid lines without markers. The S-N curves for UD 10, 15, 30° have solid, dash and short dash dot lines, respectively. The vertical axis represents the stress magnitude (the absolute value). The horizontal axis is the number of loading cycles in the logarithmic scale.

The Figure 9 shows that the FDM predictions are close to the experimental S-N curves for *R* = 0.1. Hashin and Tsai–Hill criteria predict early failure. The maximum stress criterion demonstrated overdue failure. The results are more accurate for UD 10 and 30°, but out-of-true for UD 15°.

The comparison between the S-N curves for *R* = 10 is shown in Figure 10. It indicated less resemblance for *R* = 10 than for *R* = 0.1. Too high and too low stresses were predicted by the all-failure criteria for 10° and 30°, respectively. The failure stresses have 15% difference. However, predictions for UD 15° were more accurate than for *R* = 0.1.

Figure 11 illustrates a comparison between S-N curves in combined tension-compression mode, R_crit_ = −0.91, −1.11 and −1.62 for 10°,15°, and 30°, respectively. Hashin and Tsai–Hill criteria provided conservative results and were less accurate than for the maximum stress criterion. However, this criterion indicated a tendency to overdue fatigue stress at cycles above 10^5^ for UD 10° and 15°, due to a significant variability in the degradation rate of the experimental S-N curve.

Considering a scatter of the referred data [34], the comparison indicated a good agreement between predicted and experimental results. The calculated S-N curves have a similar strength degradation rate (the S-N curves’ slope), but the magnitude of the residual strength does not always correspond to the reference data. Hashin and Tsai–Hill failure criteria demonstrate conservative results (early failure) in the most cases. The maximum failure criterion predicts precisely in some cases, but often shows overdue failure.

## 4. Discussion

The results indicated that the FDM is able to predict fatigue life of FRPs, utilizing CLDs of UD 0°, 45°, and 90°, that implies a significant reduction in required fatigue data for the model identification. 

The FDM was verified by comparison between the experimental and predicted S-N curves for UD 10°, 15°, and 30°. The verification results might be influenced by the following factors:

- The various scatter of the experimental fatigue data for different stress ratios and lay-ups that implies errors to the model identification;
- Applicability of the chosen failure criteria for a variety of lay-ups and stress states.

The experimental data and their approximation are crucial for the model identification. For example, the authors of works [40,41,42] showed the scatter of 100–1000 cycles of failure for S-N curves under various stress ratios for several FRP lay-ups. Therefore, the correct approximation becomes questionable for the limited data set for the both low- and high-cycle fatigue regimes.

There is no general failure criterion applicable for arbitrary UD FRP lay-ups under off-axis loading (which subsequently induces a multiaxial stress state), incorporating applicability limitations [43]. For the current study, Hashin and Tsai–Hill criteria provided conservative results, comparing to the maximum stress criterion for all considered stress ratios and lay-ups. Hashin and Tsai–Hill criteria take into account multiaxial stress state (normal and shear residual strengths contribute to the failure index), while the maximum stress criterion treats the strength of components independently.

The predicted S-N curves for *R* = 0.1 were more accurate than the calculated curves for other stress ratios. Moreover, the residual strength for *R* = 0.1 reduces significantly (more than 40%), and it could decrease the role of accuracy of the initial strength. Results may be improved by adjusting the failure criteria or residual strength values.

The predicted S-N curves for *R* = 10 (the compressive cyclic loading) showed less similarity with the experimental data. This is related to the dominant role of shear failure under compressive loading. The objective shear strength degradation law is difficult to determine from UD 45° tests and required additional experiments with [+45°/−45°]s lay-up [23]. Moreover, the mentioned discrepancy can be connected with extremely low strength degradation rate for *R* = 10. The S-N curves are almost flat, which increases the role of accuracy of the initial strength.

The predicted S-N curves in tension-compression for R_crit_ provide satisfactory results according to the test data. The experimental S-N curve for UD 10° has a significant non-linear behavior (even non-linear in log-scale) which causes approximation errors. Thus, the predicted stresses were more accurate for the high-cycle fatigue region than for the low-cycle one. Other accuracy issues are similar to *R* = 0.1 and *R* = 10 cases and may be related to the scatter of the experimental data.

## 5. Conclusions

In this work, the stress ratio dependent FDM was developed and verified based on published data, with the following conclusions drawn:

- Comparison between the reference fatigue data (nine S-N curves of UD 10°, 15°, and 30°) and the predictions showed a resemblance. Therefore, the hypothesis assumed was valid for fatigue life predictions of FRP laminates with those lay-ups;
- The failure criteria influence the predicted number of loading cycles, but the strength degradation rate depends only on the input approximated S-N curves. Tsai–Hill and Hashin failure criteria provide conservative predictions in the most cases;
- The FDM is able to estimate the fatigue life under arbitrary non-constant stress ratios and various stress magnitudes.

We introduced the stress ratio dependent FDM based on a hypothesis that residual strengths and stiffness reduce according to the input CLDs. The power law was used for the approximation of the experimental S-N curves. The FDM calculates the maximum number of loading cycles, failure mode, residual strength, and stiffness. The results of the present study showed the possibility of applying a phenomenological approach, excluding the Pfanner’s energy hypothesis, the applicability of which generally requires comprehensive theoretical and experimental studies.

Additional experimental CLDs for FRPs with arbitrary lay-ups will help to improve the FDM, and, therefore, it is the subject of further experimental work. In particular, the stiffness degradation law and the first-ply failure criteria implemented in the FDM need to be verified for various FRPs and lay-ups typically used in engineering applications.

## Figures and Tables

**Table 1:** The T700S/2592 sample dimensions [34].

Type of a Sample	The Long Sample, According to JIS K7076 [35]	The Short Sample, According to JIS K7083 [36]
t (thickness) [mm]	3.1	3.1
W (width) [mm]	20	10
L (length) [mm]	200	100
L_gage_ (gage length) [mm]	100	10

**Table 2:** Elastic properties of T700S/2592 [34].

Young’s modulus along X [GPa]	132
Young’s modulus along Y [GPa]	10.3
Young’s modulus along Z [GPa]	10.3
Poisson’s ratio XY	0.25
Poisson’s ratio XZ	0.25
Poisson’s ratio YZ	0.38
Shear modulus XY [GPa]	6.5
Shear modulus XZ [GPa]	6.5
Shear modulus YZ [GPa]	3.9

**Table 3:** The ultimate stresses of T700S/2592 [34].

Tensile strength along X [MPa]	1860
Tensile strength along Y [MPa]	31
Tensile strength along Z [MPa]	31
Compression strength along X [MPa]	800
Compression strength along Y [MPa]	155
Compression strength along Z [MPa]	155
Tensile strength of UD 45° (pseudo shear) [MPa]	61
Compression strength UD 45° (pseudo shear) [MPa]	150.5

**Table 4:** The fitting parameters of the Equation (3) for the input S-N curves.

Lay−up	R	A	B	C
UD [0°]	0.5	1529	−0.0219	364.1
	0.1	2180	−0.03561	−265.5
	−1	845.5	−0.03549	−47.58
	−0.43 (R_crit_)	1710	−0.0826	146.3
	10	2322	−0.004905	−1529
	2	−101.8	0.04086	891.3
UD [45°]	0.5	−27.98	0.03497	90.81
	0.1	−70.35	0.02127	132.4
	−1	326.7	−0.008352	−261.2
	−2.46 (R_crit_)	107	−0.04319	−36.06
	10	−14.87	0.05726	166
	2	−9.357	0.05288	160.3
UD [90°]	0.5	33.42	−0.02427	−2.161
	0.1	35.76	−0.04479	−4.292
	−1	33.31	−0.06944	−0.483
	−5.12 (R_crit_)	33.22	−0.0948	−0.3134
	10	−31.23	0.02087	182.1
	2	−47.35	0.009497	197.6

**Table 5:** The failure criteria used in the FDM [38,39].

Failure Criterion	Equations
Hashin	$\left\{\begin{array}{c} \frac{\sigma_{1}^{2}}{X^{2}}+\frac{\tau_{12}^{2}}{S_{L}^{2}}<1\\ \frac{\sigma_{2}^{2}}{Y^{2}}+\frac{\tau_{12}^{2}}{S_{L}^{2}}<1\\ \frac{\sigma_{2}^{2}}{4S_{T}^{2}}+\left(\frac{Y_{C}^{2}}{4S_{T}^{2}}-1\right)\frac{\sigma_{2}}{Y_{C}}+\frac{\tau_{12}^{2}}{S_{L}^{2}}<1 \end{array}\right.$
Tsai–Hill	$\frac{\sigma_{1}^{2}}{X^{2}}+\frac{\sigma_{2}^{2}}{Y^{2}}-\frac{\sigma_{1}\sigma_{2}}{X^{2}}+\frac{\tau_{12}^{2}}{S_{L}^{2}}<1$
The maximum stress	$max\left(\frac{\sigma_{1}}{X},\frac{\sigma_{2}}{Y},\frac{\tau_{12}}{S_{L}}\right)<1$

## Data Availability

Not applicable.
